# Importance of personality and coping expectancy on patient-reported hearing disability, quality of life and distress level: a study of patients referred to an audiology service

**DOI:** 10.1186/s12955-021-01802-z

**Published:** 2021-06-22

**Authors:** Øyvind Nordvik, Peder O. Laugen Heggdal, K. Jonas Brännström, Anne Kari Aarstad, Hans Jørgen Aarstad

**Affiliations:** 1 Faculty of Health and Social Sciences, Western Norway University of Applied Sciences, Bergen, Norway; 2 Department of Clinical Medicine, Faculty of Medicine, University of Bergen, Bergen, Norway; 3 Department of Otolaryngology/Head and Neck Surgery, Haukeland University Hospital, Bergen, Norway; 4 Department of Clinical Science, Section of Logopedics, Phoniatrics and Audiology, Lund University, Lund, Sweden; 5 Faculty of Health, VID Specialized University, Bergen, Norway

## Abstract

**Purpose:**

According to the World Health Organization (WHO), hearing loss (HL) affects up to 15% of the world’s adult population. Coping and personality are hypothesized to underlie quality of life (QoL) and distress scores. We aimed to study the importance of personality and coping in persons with HL for self-reported hearing disability, QoL, and distress.

**Methods:**

A cross-sectional survey was carried out, including one hundred and fifty-eight adults seeking hearing aids. Pure-tone average hearing thresholds (PTA) were determined for each ear. A revised version of the Abbreviated Profile of Hearing Aid Benefit (APHAB) served as a measure of self-reported hearing disability. Further, the generic part of the European Organization for Research and Treatment (EORTC) QoL questionnaire and the General Health Questionnaire (GHQ) (distress measure) were answered. Levels of neuroticism and the Theoretically Originated Measure of the Cognitive Activation Theory of Stress (TOMCATS) coping expectancy were determined.

**Results:**

Hearing disability was determined by PTA (better ear) and level of neuroticism. Distress and QoL were determined by neuroticism and coping.

**Conclusion:**

More neuroticism was associated with worse outcome for the variables hearing disability, distress, and QoL. Helplessness and hopelessness were associated with worse hearing disability, increased distress, and lowered QoL. Patient reported hearing disability was also associated with PTA (better ear). There is a need to investigate further the associations between hearing disability and QoL to psychosocial parameters.

## Introduction

According to the World Health Organization (WHO), hearing loss (HL) affects up to 15% of the world`s adult population, with 5.3% having disabling HL (> 40 dB HL in the better ear). The prevalence and severity of HL increases with age, mostly because of age-related HL, referred to as presbyacusis [1, 2].

HL in adults is usually assessed by pure-tone audiometry. It is, however, also recommended to ask the patient about their self-perceived degree of hearing disability. This is most often done by a few anamnestic questions, but it can also be done using questionnaires. Using such questionnaires provides more stringent measurements, and it could be argued that they should be used more frequently than presently, especially since they are even included in the European standard for “Services offered by hearing aid professionals” (EN 15927:2010). One way to assess the self-perceived degree of hearing disability is by administrating a shortened and revised version of the Abbreviated Profile of Hearing Aid Benefit (APHAB) [3], where the patient makes self-ratings of the limitations that HL has on everyday communication. To the best of our knowledge, this is currently the only validated questionnaire available for this purpose in Norwegian [4].

Patient-reported outcome measures (PROMs), including measures of patient-reported quality of life (QoL) are important when assessing both the primary consequences of a disability and the outcome of treatment and rehabilitation [5]. It has been stated that HL may be followed by serious psychosocial consequences with lowered mood and QoL [6]. Presbyacusis has been connected to both cognitive decline and depression. The mechanisms behind these associations remain unclear. Nevertheless, addressing the highly prevalent and undertreated condition of age-related hearing loss could reduce the risk of these serious diseases as well as other serious psychosocial consequences [7]. We and others have shown limited group effects of HL on QoL [8]. One reason for this might be that QoL scores primarily depend on psychosocial variables and secondarily on the disability directly caused by a specific disease, as previously seen for head and neck cancer [9]. Still, as QoL scores vary among persons with HL, it is important to understand the influence of psychosocial causes on this variation. It is furthermore not clear if ratings of self-perceived degree of hearing disability, as assessed using questionnaires such as the revised APHAB, are related to psychosocial factors at the individual patient level, as previously shown for QoL and distress questionnaires [10].

The US National Comprehensive Cancer Network defines psychological distress as “an unpleasant emotional experience of a psychological, social, or spiritual nature”. Distress extends along a continuum, ranging from common normal feelings of vulnerability, sadness, and fear to problems that can become disabling, such as depression, anxiety, panic, social isolation, and spiritual crisis [11]. Within psychological research, distress is often quantified as the sum of anxiety and low mood [12]. The level of distress may be measured by the general health questionnaire (GHQ) [13]. This questionnaire measures the relative emotional state of the subject during the last two weeks. A high GHQ score has been proposed as an indicator of mental disease and is regarded as the sum of anxiety and depression [13]. HL is a risk factor for distress [2] and furthermore, the score of GHQ correlates to QoL scores [10]. Therefore, it is of interest to study distress associated with HL, both directly and as associated with pertinent psychosocial factors.

There are several psychosocial factors that may potentially influence QoL measures. These are, for example, stressors and individual personality traits. Selye [14] related stress to the response to the strain, rather than to the source of strain. For the present purposes, a disability or a disease like HL can be strain in life. He used the term “stressor” to denote the source of strain, stressors are, however, not necessarily something that one would avoid [15]. Indeed, too limited stressor exposure may also have negative cognitive consequences [16]. Thus, when facing strain in life, such as the condition of HL, how we employ available coping strategies may be more important than the strain itself for QoL levels [15]. One recent approach in studies of psychological coping is to focus on coping expectancy. This can be studied based on the cognitive activation theory of stress (CATS). A short questionnaire based on the CATS theory has been developed and named the theoretically originated measure of the CATS (TOMCATS). This aims to measure “positive” (active), “negative” (hopelessness), and “no” (helplessness) coping expectancy. We have used this questionnaire to study the expected choice of coping, an underlying measure in PROMs scores, among persons with HL.

Personality may be defined as those characteristics of a person that account for consistent patterns of feelings, thinking, and behavior [9, 17]. Personality traits refer to internal characteristics that are presumed to predict behavior [18] and relate to unique individual characteristics [19]. Neuroticism is a personality dimension which refers to experiencing dysphoric emotional states [9, 17]. While low neuroticism predicts low stress and threat appraisal, high neuroticism predicts high stress and threat appraisal [20]. Neuroticism is about 50% genetically generated [21] and has been shown to affect QoL [22], distress [23], and to interact with the choice of coping [24]. To understand the relationship between distress caused by HL and QoL, it is necessary to include measures of the consequences of neuroticism, as well as on the reported choices of coping.

The relationships between personality traits, choice of coping, and QoL have been studied among patients with cochlear implants (CI). Muigg and colleagues [25] found that hearing related QoL improved in patients receiving CI, and furthermore, the QoL scores were affected by neuroticism. Cox et al. have reported that personality traits seem to be associated with reported hearing problems both before and after the fitting of hearing aids (HA). Porter and Boothroyd have furthermore shown similar findings in patients with hearing loss due to Meniere’s disease [26].

QoL in individuals with HL thus seems to represent a complex measure. Individual variation in QoL may be constructed from personality traits, i.e., neuroticism and choice of coping. These factors may account for some of the QoL variation seen in different respondent groups [27], including persons with HL [28]. We suggest that an axis of causality, from personality, via the choice of coping to hearing disability may be present in persons with hearing loss.

Hence, the main aims of this investigation were to investigate any relationship between hearing disability, pure-tone audiometry, QoL, distress, choice of coping, and personality among subjects with HL referred for first time fitting or renewal of hearing aids (HAs). We hypothesize that QoL, hearing disability and distress in persons with HL are associated with the level of neuroticism and with coping expectancy.

## Materials and methods

Three hundred and one patients were invited to participate in the study by letter before a planned consultation. Both first-time HA users, and patients referred for HA renewals were invited. Invited subjects were adults (age 18–78 years) with HL judged in need for HA fitting at Haukeland University Hospital located in western Norway. In Norway, citizens with hearing loss have a legal right to two hearing aids every six years. Costs of the hearing aids and repairs are covered by the government. One hundred and fifty-eight patients returned the questionnaires (response rate of 52.5%); Table 1 shows demographic and descriptive information of included patients. The Norwegian Regional Committees for Medical and Health Research Ethics approved the project (project reference: 2013/1302).

**Table 1:** Descriptive Information for included subjects

		Range
Age (mean ± SD years)	61 ± 10	27–78
Males/females (N)	96/62	
Type hearing loss (N)		
Sensorineural	144	
Mixed	13	
Diagnosis (N)		
Presbyacusis	42	
Noise induced	49	
Hereditary	20	
Congenital	12	
Meningitis	2	
Sudden hearing loss	7	
Middle ear disease	11	
Other trauma	4	
Vestibular schwannoma	6	
Unknown	2	
SUM	158	
Pure-tone average for frequencies (PTA) 0.5, 1, 2, 4 kHz		
Better ear (mean ± SD)	34 ± 17 dB HL	2.75–107 dB HL
Worse ear (mean ± SD)	45 ± 19 dB HL	3.75–112 dB HL
First time users (N)	87	
Duration hearing loss (years)		
(mean ± SD)	22 ± 18	0–76

Demographic data and information regarding diagnosis and pure-tone audiograms were collected from medical records. Audiograms were obtained during visits to the clinic, and all equipment was calibrated according to ISO-389-1 [29]. The patients were grouped by better ear pure-tone average (PTA; frequencies 0.5, 1, 2 and 4 kHz) as having normal hearing (< 26 dB HL), slight/mild hearing loss (26–40 dB HL), moderate hearing loss (41–60 dB HL) or severe to profound hearing loss (61 dB HL and more). Sensorineural hearing loss was defined by an average air-bone-gap (0.5, 1, 2 and 4 kHz) ≤ 10 dB HL. A conductive hearing loss was identified by an average air–bone gap ≥ 15 dB HL and average bone-conduction thresholds ≤ 25 dB HL (0.5, 1, 2 and 4 kHz). Average bone-conduction thresholds worse than 25 dB HL in combination with average air-bone gaps ≥ 15 dB HL were categorized as a mixed hearing loss (0.5, 1, 2 and 4 kHz). Bilateral (*n* = 135) or unilateral HL (*n* = 23) was determined. A better ear with pure-tone hearing thresholds equal to or better than 25 dB HL at octave frequencies 0.125–8 kHz and a worse ear with a PTA ≥ 25 dB HL defined unilateral hearing loss. Experienced hearing aid users (*n* = 71) were currently fitted with a variety of HAs, in-the-ear (11.1%), behind-the-ear (80%), or bi-CROSS systems (8.9%). A similar distribution of fittings was planned for the first-time HA users (*n* = 87), with 19% in-the-ear, 76.2% behind-the-ear, and 4.8% bi-CROSS systems.

### Questionnaires/instruments

An overview of all included questionnaires is given in Table 2.

**Table 2:** Questionnaires applied in the study. Scoring, min and max scores, mean scores and standard deviations, as well as the Cronbach’s α for total scales and sub-scales

	Questionnaire (*Subscales*)	Abbreviation	Items	Scoring	Exemplary item	**Min**	**Max**	**Mean (SD)**	*α*
Outcomes	**Abbreviated profile of hearing aid benefit**	APHAB	12	Four-point Likert format (Higher is better)	*"I have to ask people to repeat themselves in one-on-one conversations in a quiet room"*	1	3.92	2.63 (.70)	.93
	*Communication in quiet conditions*		7			1	4	2.86 (.72)	.89
	*Communication in adverse conditions*		5			1	3.8	2.3 (.78)	.89
	**European Organization for research and treatment core quality of life questionnaire**	EORTC QLQ-C30	17						
	*General quality of life*		2	Seven-point Likert format (Higher is better) *	*"How has your quality of life been the past week"*	0	100	73.6 (21.26)	.91
	*Functional quality of life*		15	Four-point Likert format (Higher is better) *	*"Have you had difficulties going for a short walk outside?*	33	100	85.81 (15.1)	.87
	**General health questionnaire**	GHQ-12	12	Four-point Liker format (Higher means more distress)	*"Have you been able to focus completely on what you have been doing?"*	1.17	3.08	1.82 (.28)	.81
Predictors	**Eysenck personality inventory**	EPI	23	Yes or no (Higher score means more neuroticism)	*"Do you worry about your health?"*	0	19	6.97 (5.04)	.80
	**Theoretically originated measure of the cognitive activation theory of stress**	TOMCATS	7						.75
	*Positive Coping*		1	Four-point Likert format (Higher score means more coping)	*"I can solve most difficult situations with a good result"*	1	4	3.39 (.60)	
	*Hopelessness*		3	Four-point Likert format (Higher score means more hopelessness)	*"All my attempts at making things better just make them worse"*	1	3.33	1.32 (.56)	.78
	*Helplessness*		3	Four-point Likert format (Higher score means more helplessness)	*"All my attempts at changing my life are meaningless"*	1	3.67	1.57 (.66)	.77

*Transformed so that 100% indicates best function and 0% indicates worst function

### Abbreviated profile of hearing aid benefit (APHAB) (revised version)

As a measure of hearing disability, the present study utilized a revised and shortened version of the Norwegian translation of APHAB that examines self-assessed communication ability in both quiet and adverse listening conditions[3]. The questionnaire consisted of twelve items extracted verbatim from the APHAB subscales “ease of communication” and “background noise”. Each item was scored using a revised four-point Likert format: “always/most of the time” (1), “half the time” (2), “sometimes” (3), and “never/very rarely” (4). A mean sum score for the total scale was calculated, and mean sum scores for the subscales “communication in quiet conditions” (items 2, 5, 6, 7, 8, 11 and 12) and “communication in adverse conditions” (items 1, 3, 4, 9 and 10) [4]. All subjects reported their unaided communication ability. Higher scores mean less communication difficulties.

### EORTC QLQ quality of life measure (EORTC QLQ-C30)

The general QoL was assessed using the European organization for research and treatment (EORTC) core quality of life (QLQ-C30) questionnaire [30]. From the QLQ-C30, the global health and QoL scales and five functional scales (physical, role, cognitive, emotional, and social) were reported. The answers were given according to a four-point Likert format, except for questions about general health and QoL, which were given according to a seven-point Likert format. All responses were scored in accordance with the EORTC Scoring Manual. The C30 functional scales and the global scale were transformed so that 100% indicated the best function and 0% the worst function. A “functional” sum score was calculated from the five functional scales.

### General health questionnaire (GHQ-12)

The general health questionnaire (GHQ)-12 was used to assess the level of distress [11]. This questionnaire was used to assess a subject’s current state and whether there is a difference from the subject’s usual state. This was scored according to a standard 4-point response Likert matrix referring to the last two weeks: (1) better than usual, (2) as usual, (3) worse than usual, (4) much worse than usual. Higher scores mean more distress.

### The theoretically originated measure of the cognitive activation theory of stress (TOMCATS) questionnaire

The theoretically originated measure of the cognitive activation theory of stress (TOMCATS) questionnaire was used to measure the degree of general response outcome expectancies as described in the cognitive activation theory of stress (CATS) [15]. This questionnaire consisted of seven items [31]. Answers were given according to a 4-point Likert format, rated from 1 (not true at all) to 4 (completely true) [31]. Three outcome expectancy dimensions were measured, and categorized as either positive coping (one item, higher score means more positive coping), hopelessness (three items, higher score means more hopelessness), or helplessness (three items, higher score means more hopelessness) [31].

### Eysenck personality inventory (EPI)

The neuroticism dimension of the Eysenck personality inventory was determined [32]. The neuroticism scale, consisting of 23 questions, assessed adjustment versus emotional instability and identifies individuals prone to psychological distress and maladaptive coping responses. The scale included questions related to mental symptoms (obsessive thoughts, anxiety, depression, and low self-esteem) and somatic symptoms (muscle pain, tachycardia, and sleeplessness). Items were scored “yes” (1 point) or “no” (0 points) and calculated as sum scores. A higher score indicated more neuroticism. Persons with low scores are characterized as relaxed, unemotional and calm [33].

### Data management and analysis

Patients completed the questionnaires by pen and paper at home, and the questionnaires were returned by regular mail. Questionnaires were typically mailed to patients three to four weeks before the hearing aid fitting, and they were encouraged to return the questionnaires within one week. Thus, the data were collected within one month before the hearing aid fitting. For questionnaires that use mean sum scores, scores were not calculated if single items were missing.

Statistical analyses were performed using the statistical program package IBM SPSS (Ver. 25.0 for Windows; IBM Corp. Armonk, NY, USA). Associations between variables were investigated using Pearson product-moment correlation coefficients (Pearson`s r) and partial correlations. The Fisher r-to-z transformation applied a value of z to assess the significance of the difference between the correlation coefficients (in Table 5).

Prediction of outcome was assessed using stepwise linear regression. For missing values, cases were excluded pairwise. As stepping method criteria, the probability of F was used (entry 0.05 and removal 0.1). The linearity of the relationship between dependent and independent variables was tested with a bivariate plot of the standardized residuals and standardized predicted values. Then, by fitting a Loess curve through the scatterplot, it was ensured that the relationship between standardized predicted and residuals was linear around zero. Further, the normality of residuals was tested by comparing the observed cumulative distribution function (CDF) of standardized residuals to the expected CDF of a normal distribution, using a P–P plot. Additionally, the observed quantile and the theoretical quantile of a normal distribution of residuals was assessed using a Q–Q plot. No variables were transformed prior to the regression analysis. Variables were checked for collinearity. The presence of collinearity was defined as the combination of a value of tolerance < 0.2 and a variance inflation factor (VIF) > 10. Variables from the models revealed by the stepwise procedure were then directly entered in models that also included the variables sex and age. Both the corrected and uncorrected models are presented in Table 4.

## Results

### Demographic variables

One hundred and fifty-eight adult patients (27–78 years old) with a mean age of 61 years participated in this study. The sample consisted of 62 females and 96 males. Seventy-one (45%) of the participants were experienced HA users seeking HA renewal, while the others (n = 87) (55%) were first time HA users. Years of living with a HL ranged from 0 to 76 years, with a mean of 22 years (*n* = 126, SD ± 18). Most of the included patients suffered from sensorineural HL (n = 145), while 13 patients had mixed HL (Table 1).

In patients with HL, age did not correlate to functional QoL scores, except for the “role” functional scale. This scale comprises aspects of occupational and social roles. Hearing disability scores were not associated with age in patients. Males and females showed QoL scores at equal levels except for physical QoL where males reported better scores than females.

### Correlations

Correlations between PTA for better and worst ear, inter-aural difference in PTA (*M* = 11.36, *SD* = 15), APHAB scores, TOMCATS scores, level of neuroticism, GHQ score, EORTC scores, age and duration of hearing loss are shown in Table 3.

**Table 3:** Pearson’s product-moment correlations

				PTA			APHAB			QoL				Coping expectancy	
		Age	HL duration	Worst	Better	Inter-aural difference	Total	Quiet	Adverse	General	Functional	GHQ	Neuroticism	Positive Coping	Helplessness
	HL Duration	− .04													
PTA	Worst	− .01	.34**												
	Better	.14	.41**	.65**											
	Inter-aural difference	− .16	.04	.51**	− .31**										
APHAB	Total	− .02	− .24**	− .31**	− .38**	.05									
	Quiet	− .07	− .26**	− .28**	− .36**	.07	.95**								
	Adverse	.04	− .21*	− .30**	− .34**	.02	.91**	.74**							
QoL	General	.15	− .17	− .05	.03	− .14	.26**	.22**	.27**						
	Functional	.07	− .11	− .02	.03	− .14	.31**	.28**	.31**	.79**					
	GHQ	− .13	.14	− .11	− .21*	.11	− .18*	− .17*	− .17*	− .50**	− .44**				
	Neuroticism	− .28**	.07	− .03	− .13	.08	− .19*	− .19*	− .17*	− .47**	− .46**	.56**			
Coping expectancy	Positive Coping	.04	− .15	− .08	− .05	− .1	.26**	.24**	.25**	.42**	.34**	− .39**	− .41**		
	Helplessness	.001	.06	− .05	− .08	− .1	− .17*	− .15	.17*	− .55**	− .44**	.40**	.47**	.52**	
	Hopelessness	− .10	.09	− .02	− .10	− .17	− .14	− .11	− .11	− .38**	− .37**	.34**	.41**	− .44**	.72**

**Correlation is significant at the 0.01 level (2-tailed)

*Correlation is significant at the 0.05 level (2-tailed)

*HL* Hearing loss, *PTA* Pure-tone average, *APHAB* Abbreviated profile of hearing aid benefit, *QoL* Quality of life, *GHQ* General health questionnaire

### Prediction of outcome

APHAB scores, GHQ-12 scores and EORTC-scores were subsequently subject to stepwise linear regression analyses as dependent variables, including TOMCATS score, neuroticism and the PTA in the better and worst ear and duration of hearing loss, and the inter-aural difference in PTAs as the independent variables. Corrected models included variables from the stepwise procedure, age and sex.

Worse hearing disability was associated with poorer hearing and more neuroticism. More distress was associated with more neuroticism, less coping and poorer hearing. Better general QoL was associated with less helplessness, less neuroticism and more positive coping. Better functional QoL was associated with less neuroticism and less helplessness (Table 4).

**Table 4:** Stepwise linear regression models of APHAB, GHQ and EORTC and models corrected for sex and age

Uncorrected				Corrected for sex and age				
Model		Std. Beta	R^2^ change		Sig	*Std. Beta*		*Sig*
APHAB								
2	*PTA best ear*	− .38	0.13		.000	*PTA best ear*	*− .41*	*.000*
	*Neuroticism*	− .28	0.08		.000	*Neuroticism*	*− .25*	*.002*
						*Age*	*− .03*	*.66*
						*Sex*	*.02*	*.84*
GHQ								
3	*Neuroticism*	.47	0.34		.000	*Neuroticism*	*.47*	*.000*
	*Positive Coping*	− .46	0.04		.003	*Positive Coping*	*− .21*	*.005*
	*PTA best ear*	− .19	0.04		.003	*PTA best ear*	*− .16*	*.020*
						*Age*	*.02*	*.77*
						*Sex*	*− .06*	*.40*
General QoL (EORTC)								
3	*Helplessness*	− .29	0.26		.000	*Helplessness*	*− .35*	*.000*
	*Neuroticism*	− .58	0.08		.000	*Neuroticism*	*− .38*	*.004*
	*Positive Coping*	.17	0.02		.040	*Positive Coping*	*.14*	*.044*
						*Age*	*.07*	*.29*
						*Sex*	*− .08*	*.23*
Functional QoL (EORTC)								
2	*Neuroticism*	− .33	0.21		.000	*Neuroticism*	*− .33*	*.000*
	*Helplessness*	− .30	0.08		.000	*Helplessness*	*.29*	*.000*
						*Age*	*− .02*	*.76*
						*Sex*	*− .08*	*.29*

**Uncorrected** = Independent variables: TOMCATS, Neuroticism, PTA in better and worse ear, duration of hearing loss and inter-aural difference in PTA

**Corrected** = Models from the stepwise regression corrected by directly entering the variables in a model that also included age and sex as independent variables

### Scores dependent of specific diagnosis

APHAB scores, EORTC scores, GHQ-12 scores, TOMCATS scores, and neuroticism level did not differ between people diagnosed with different types of hearing loss (results not shown). To investigate whether the relationship between coping and QoL was affected by the time of hearing loss onset, we investigated whether correlations between coping and QoL had a statistically significant higher common variance in patients with congenital or hereditable disease compared to patients with presbyacusis or noise-induced HL. These correlations were statistically significantly stronger among the patients with hereditary and congenital causes for their HL versus patients with presbyacusis or noise-induced HL (Table 5).

**Table 5:** Pearson`s product-moment correlation coefficients between coping expectancies and general and functional quality of life in those with Presbyacusis or noise-induced hearing loss and those with hereditary of congenital hearing loss

Diagnosis	Presbyacusis and noise-induced (n = 91)			Hereditary and congenital (n = 32)		
TOMCATS	Positive Coping	Helplessness	Hopelessness	Positive Coping	Helplessness	Hopelessness
*QoL*						
General	.32**	− .49**	− .29**	.78**	− .79**	− .75**
Functional	.25*	− .38**	− .34**	.56**	− .69**	− .59**

*p < 0.05; **p < 0.01; ***p < 0.001

Bold written coefficients: Statistically significant stronger correlation coefficient in those with hereditary or congenital cause of hearing loss than in those with presbyacusis or noise-induced hearing loss

### First time users and experienced hearing aid users

Table 6 shows descriptive information for age, duration of hearing loss and PTA in better and worse ear as well as APHAB, EORTC, GHQ, Neuroticism and TOMCATS scores for those referred for their first hearing aid fitting, and for experienced hearing aid users. A one-way MANCOVA was used to compare hearing disability, QoL, distress, neuroticism and coping between the groups. Age, duration of hearing loss and the PTA from the better and worse ear were included as covariates. There was no statistically significant difference between the groups on the combined dependent variables after controlling for the covariates, *F*(9, 97) = ,87^b^, *p* = 0.56, Wilks` Λ = 0.93, partial η^2^ = 0.075.

**Table 6:** Descriptive information and scores for first time- and experienced hearing aid users

	*M* (SD)		
	First time users	Experienced users	*p*^a^
Age	60.24 (9.38)	61.82 (11.02)	.33
Duration	16.5 (16.4)	28.8 (16.6)	**< .001**
PTA best	26.24 (12.80)	43.45 (17.44)	**< .001**
PTA worse	37.63 (15.62)	53.96 (19.11)	**< .001**
APHAB			
Total	2.78 (.63)	2.44 (.74)	
Quiet	3.0 (.65)	2.69 (.78)	
Noise	2.47 (.73)	2.08 (.77)	
EORTC			
General	74.03 (20.79)	72.70 (21.95)	
Functional	85.88 (15.48)	85.76 (14.65)	
GHQ	1.84 (.28)	1.79 (.27)	
Neuroticism	7.13 (5.04)	6.76 (5.00)	
TOMCATS			
Positive coping	3.4 (.56)	3.37 (.64)	
Helplessness	1.57 (.70)	1.57 (.64)	
Hopelessness	1.31 (.54)	1.34 (.58)	

^a^Independent samples *t*-tests. Significant differences between groups (corrected for multiple comparisons using Bonferroni correction) are set in bold

## Discussion

This study aimed to investigate whether poorer hearing, maladaptive coping expectancies and more neuroticism were associated to poorer QoL, worse hearing disability and more distress in a population of HA users and HA candidates. We have shown that hearing disability, QoL, and distress were associated with each other and to coping expectancies. More neuroticism was associated to poorer QoL, worse hearing disability and more distress.

As previously stated, the personality dimension neuroticism is a broad pervasive dimension of normal personality whereby people vary in their tendency to experience dysphoric emotional states [32, 33]. Furthermore, neuroticism inversely predicts QoL [24, 27] among various patient groups. Such a relation is presently supported also among persons with HL both regarding QoL and hearing disability.

Variable coping may have secondary consequences for QoL [24, 27]. Presently, it has been shown that positive coping was positively associated with QoL, whereas helpless and/or hopeless were negatively associated with QoL. Less hearing disability was also associated with positive coping expectancies. This supports that hearing disability may be viewed as symptom specific QoL. The association between HL and choice of coping may be a consequence of adaption to long-term HL, and thus the coping expectancy could be a consequence of the HL. This should be studied more closely.

Some previous studies have suggested gender differences for coping with hearing loss. No such differences were seen in the present study. We suggest that this discrepancy could partly be explained by the type of coping-measure applied. The TOMCATS questionnaire used in this study is a measure of general coping expectancy. Thus, the questions are not related to how the subject copes with hearing loss and/or communication. Studies that have suggested gender differences have used questionnaires that specifically address coping with hearing loss while others have interviewed patients and specifically addressed coping with hearing loss [34]. The discrepancy between general and hearing-specific measures of coping should be addressed directly in later studies.

We have not found any statistically significant relationship between age or gender and QoL, hearing disability or coping in the present cohort. Hearing disability, QoL, and distress were not related to demographic variables. Some previous studies have shown that the discrepancy between hearing disability and pure-tone hearing threshold measures vary with age [35, 36]. This is suggested to be related to factors such as an increased acceptance of hearing loss in older adults, less demanding communication needs and the level of perceived stigma [35]. Results of the present study did not reveal any significant correlation between hearing disability and age. While the reasons for the effect of age on the association remains uncertain, a simple correlation between PTAs and self-reported hearing disability is not expected over a large age range. We suggest that this association could even be affected by the type of self-report tool used. Hence, asking “do you have trouble hearing” could yield different correlations to PTAs than asking more detailed questions on communication abilities in various listening scenarios. We did not investigate the discrepancy between hearing disability and PTAs over age-groups, but this could be addressed in future studies that apply the revised APHAB used in this study.

We have previously shown that hearing disability shares a common variance with PTA in the better ear at about 14%. However, hearing disability was associated not only with pure-tone-audiometry, but also with the degree of neuroticism and with coping expectancy with combined common variances to these two parameters at about the same percentage as to PTA. Furthermore, hearing disability correlated to QoL, but a significant association could not be found by multiple stepwise regression analyses, including a wide array of parameters. Taken together, the present measure of hearing disability seems to behave as expected for a symptom specific QoL questionnaire. Regarding QoL, the results support that QoL in persons with HL is constructed as in other patients groups [37].

We have shown that among patients with HL due to hereditary or congenital causes, i.e., patients with early-onset HL, the associations between coping expectancies and QoL scores were significantly stronger compared to patients with other causes of HL. This is in line with previous observations among patients with challenging disease consequences [24, 38]. This could indicate that patients with early-onset HL should be closely monitored by healthcare services.

We have a limited number of respondents. Thus, any lack of significant differences between groups should be interpreted with caution. This investigation did not include the elderly above 80 years of age with hearing loss. Many investigations regarding HL have focused on this group, and the present results should not be extrapolated to such age groups without taking appropriate precautions. Furthermore, the present response rate is limited. The strong correlations, however, between measures of psychological factors and QoL scores add validity to the findings of this study. In a broader sense, the principally equivalent results have been presented in previous studies regarding other disabilities, and these results support that QoL seen in persons with HL is based on psychosocial interactions also seen in other patient groups [39–42].

Results from the present study suggest that an axis of causality, from personality, via the choice of coping to hearing disability, may be present. However, the direction of causality cannot be determined by the present cross-sectional design. Nevertheless, this may be taken into consideration when interpreting hearing disability at the individual patient level in clinical settings. It could, for example, be suggested that individuals that demonstrate large discrepancies between hearing disability and pure-tone audiometry and tests of speech perception could be affected by adverse psychosocial factors [39–42]*.* In such cases, the treatment could be adjusted to allow a broader approach to the therapy, with an increased focus on individual counselling and psychosocial therapy.

## Conclusion

We have shown that more neuroticism was associated with worse outcome for the variables hearing disability, distress, and QoL. Helplessness and hopelessness were associated with worse hearing disability, increased distress, and lowered QoL. Further, the present study showed that more aspects than previously generally recognized must be taken into consideration when evaluating both hearing disability and QoL scores in persons with HL. Patients with early-onset HL presumably should be closely followed up. Further studies are needed as to how and when psychosocial matters should be taken into special consideration.

## Data Availability

According to local laws and regulations, and the approvals given from authorities in advance of performing this study, data and materials cannot be shared or made available.
